# A clinical evaluation of the TK 210 ELISA in sera from breast cancer patients demonstrates high sensitivity and specificity in all stages of disease

**DOI:** 10.1007/s13277-016-5024-z

**Published:** 2016-04-14

**Authors:** J. Kiran Kumar, A. C. Aronsson, G. Pilko, M. Zupan, K. Kumer, T. Fabjan, J. Osredkar, S. Eriksson

**Affiliations:** 1 Department of Anatomy, Physiology and Biochemistry, Swedish University of Agricultural Sciences, VHC, PO Box 7011, SE 75007 Uppsala, Sweden; 2 AroCell AB, Virdings Allé 32B, SE-754 50 Uppsala, Sweden; 3 Institute of Oncology, Ljubljana, Slovenia; 4 Blood transfusion Centre, Ljubljana, Slovenia; 5 Institute of Clinical Chemistry and Clinical Biochemistry, University Medical Centre Ljubljana, Ljubljana, Slovenia

**Keywords:** Thymidine kinase 1, Sandwich TK 210 ELISA, CA 15-3, Breast cancer, Prognosis

## Abstract

Thymidine kinase (TK1) is an enzyme involved in DNA synthesis that leaks into the blood as a result of high cell turnover, particularly in the case of cancer. Serum TK1 activity has been used for prognosis and monitoring of leukemia and lymphoma patients for many years. Here, we describe the first clinical results with the newly developed TK 210 ELISA from AroCell AB. Sera from 124 breast cancer patients with known TNM classification along with sera from 53 healthy females were analyzed by TK 210 ELISA for TK1 protein and TK1 activity levels by the ^3^[H]-deoxythymidine (dThd) phosphorylation assay. The limit of detection for the TK 210 ELISA was 0.17 ng/ml, and 60 % of the sera from female blood donors were below this value. The median TK1 levels found in sera from breast cancer patients with T1 to T4 stage disease were 0.31, 0.46, 0.47, and 0.55 ng/ml, and these levels significantly differed from healthy controls. The median values of the biomarker CA 15-3 were also increased in patient sera from T1 to T4 patients (16, 34, 36, 40 U/ml, respectively). TK 210 ELISA showed significantly higher sensitivity for the T1 and T2 breast cancer patients compared to the TK activity assay. The combination of the TK1 ELISA and CA 15-3 biomarkers demonstrated a significant increase in sensitivity up to 15 % compared to each marker alone. This evaluation of the TK 210 ELISA strongly suggests that it can provide independent and complementary information for patients with breast cancer.

**Electronic supplementary material:**

The online version of this article (doi:10.1007/s13277-016-5024-z) contains supplementary material, which is available to authorized users.

## Introduction

Breast cancer is one of the most common forms of cancer that accounts for 10–18 % of all cancer deaths in women [1]. Pre-operative chemotherapy in combination with radiation or hormonal therapy has increased the efficiency of breast cancer treatment [2, 3]. Still, the anti-cancer therapy is not very effective especially in the case of patients with metastatic disease. Tumor biomarkers are the substances that indicate the presence of cancer and play an important role in patient management. Serum biomarkers are potentially useful for early diagnosis, therapy monitoring, and predicting tumor relapse [4]. Several biomarkers such as estrogen receptor (ER) [5], human epidermal growth factor receptor 2 (HER-2/neu) [6], carbohydrate antigen 15-3 (CA 15-3) [7], and carcinoembryonic antigen (CEA) [8] have been evaluated for prognosis, diagnosis, and treatment monitoring in breast cancer. Out of these, CA 15-3 is the most commonly used. It is a protein product of the MUC-1 gene, which is shed into the blood stream in an under glycosylated form from adenocarcinomas [9]. According to the ASCO 2015 guidelines, CA 15-3 and CEA can be used for monitoring the response to therapy only together with physical examination and imaging. The relatively low sensitivity and poor specificity of CA 15-3 for early stage tumors limits its use. So there is a need for biomarkers that can fulfil the dual purpose of detecting the tumors at early stages of progression and monitoring treatment response.

Thymidine kinase 1 (TK1) is another interesting serum biomarker that has been used for prognosis and monitoring therapy in leukemia and lymphoma patients during the last two decades [10–13]. TK1 is an ATP dependent enzyme involved in synthesis of pyrimidine deoxy nucleotides via the salvage pathway. The protein and activity levels of TK1 are cell cycle regulated and both increase in late G1/early S phase and reach a peak in late S/early G2 phase. Finally the TK1 protein is degraded by a specific mechanism in M-phase [14–17]. Currently, different assays are used to measure serum TK1 activity including the TK-REA, TK-Liaison, Divitum, and ^3^[H]-dThd phosphorylation assays. Studies have shown that these assays are clinically valuable for prognosis and monitoring therapy particularly in lymphoma and leukemia diseases [11–13]. Development of antibodies against different regions of TK1 to determine TK1 protein levels provided an alternative to TK1 activity assay [18, 19]. Several clinical studies with an antibody based dot blot assay demonstrated that TK1 protein assays have higher sensitivity than the TK1 activity assays in patients with solid tumors [20–23]. Another immunoaffinity assay has been described recently, and it showed similar results to other TK1 protein assays with breast and prostate cancer samples. Furthermore, differences in the molecular forms of serum TK1 in patients with different types of tumors could be identified [24]. However, these assays are not compatible with routine clinical use. Several attempts have been made to develop an ELISA test for serum TK1 quantification [25–27] but so far none of them are commercially available for clinical use. Therefore there is a need to develop a TK1 ELISA with a detection limit in the range of nanograms per milliliter.

Here we evaluate the performance of the new sandwich TK 210 ELISA produced by AroCell AB, Uppsala, Sweden, which is based on two monoclonal antibodies raised against the C-terminal region of the TK1 protein. The results were obtained with clinical samples from breast cancer patients with known TNM staging as well as CA 15-3 levels. The same samples were analyzed with TK 210 ELISA and TK1 activity assay [24, 28]. The TK 210 ELISA appears to offer many advantages such as elimination of radioactive materials, easily performed in clinical chemistry laboratories, and a capacity to detect early stage breast cancer disease.

## Materials and methods

### Serum samples

Sera from healthy females (*N* = 53) were collected at the Blood Transfusion Centre in Ljubljana, pre-treatment breast cancer sera (*N* = 76) were collected at the Institute of Oncology at Ljubljana, processed and stored at −20 °C until they were analyzed for CA 15-3 at the University Medical Centre Ljubljana as well as for TK1 activity (^3^[H]-dThd phosphorylation assay) and TK1 protein at the Swedish University of Agricultural Sciences, Uppsala. The project was approved by the Slovenian Medical Ethics committee (KME 83/04/08). Another set of breast cancer sera (*N* = 48) were purchased from Biotheme Research Solutions Inc., FL, USA. These samples were collected as de-identified diagnostic remainders exempt from Title 46, Title 21, and HIPAA IRB/Consent requirements. The serum samples were collected under an IRB-approved protocol or collected as consented donor samples from FDA-licensed/registered facility following GMPs. The mean and median age of healthy individuals was 44 years (range 22–63 years) and 62 years (range 30–92) in the case of breast cancer patients. Tumor staging was performed according to tumor size, lymph nodes, and metastasis (TNM) classification by AJCC.

### ^3^[H]-dThd phosphorylation assay

The TK1 activity in serum samples was determined by using an optimized ^3^[H]-dThd phosphorylation assay as described previously [24, 28]. In brief, 10 μl of serum sample was incubated with a reaction buffer containing 20 mM Tris/HCl, pH 7.6, 2 mM dithiothreitol, 5 mM NaF, 5 mM MgCl_2_, 5 mM ATP, and 5 μM of dThd at 37 °C for 1 h. Then 10 μl was applied on DE-81 filter paper (Whatman). The unbound substrate was removed by ammonium formate wash, and the products were eluted and the radioactivity was measured by scintillation counting [28]. TK1 activity is expressed as picomolar dTMP formed per minute and milliliter serum.

### The sandwich TK 210 ELISA procedure

TK1 protein levels in serum samples were determined using the TK 210 ELISA, following the manufacturer’s instructions (www.arocell.com). The following procedure was performed. (1) Serum samples, controls, and calibrators were pre-incubated with the sample dilution buffer for 1 h at room temperature. (2) An antibody coated 96-well plate in the kit was pre-washed 4 times with wash buffer using a Tecan Hydroflex washer before adding the incubated samples and calibrators. (3) After a pre-wash, 100 μl of calibrators, controls, and serum samples were added to each well, and the plate was incubated for 2 h on a rocking platform. (4) After non-binding molecules were washed away during 4 × 2 min with the wash buffer, 100 μl of biotin-labelled anti-TK1 antibody was applied to each well followed by incubation for 1 h. (5) Then, the plate was washed again 4 times and incubated with the streptavidin-HRP solution (100 μl per well) for 30 min. (6) A final wash with wash buffer 4 times to remove unbound HRP was done. (7) The wells were subsequently incubated for 15 min with the TMB solution. The reactions were stopped by adding 2 N HCL, and the absorbance in each well was determined using a microplate reader set to 450 nm (reference wave length 540 nm). Serum TK1 protein levels were determined using the calibrator curve from each plate and the 4-PL curve fit program. All measurements with serum samples were done in duplicates. The limit of detection (LOD), cut-off value for healthy, and the overall coefficient of variation (CV) were determined. The TK1 protein concentration (ng/ml) in each patient was calculated after adding the LOD value, i.e., 0.17 ng/ml to all values.

### The CA 15-3 analysis

Breast cancer sera from different stages were analyzed for CA15-3 using a commercial chemiluminescence assay for the Liaison analyzer (DiaSorin) as described in the product instructions. The cut-off value (29 U/ml) was estimated based on CA 15-3 levels in sera from healthy females (*N* = 53).

### The TK-Liaison assay

Sera from healthy females (*N* = 53) and breast cancer (*N* = 76) were also analyzed using the commercially available TK-Liaison assay according to the manufacture’s instructions (www.DiaSorin.com) in order to determine the correlation with the ^3^[H]-deoxythymidine (dThd) phosphorylation assay.

### Statistical analysis

The TK1 activity and TK1 protein distribution in sera from healthy and breast cancer patients were tested for normality using the D’Agostino and Pearson omnibus normality test. The TK1 activity and protein levels in healthy as well as in breast cancer patients showed non-Gaussian distribution. The spearman correlation coefficient (*rs*) was used to determine the correlation between TK1 activities and TK1 protein levels. STK1 activity, TK1 protein levels, and CA 15-3 values in different sub groups were log transformed (log10) before analysis. The Mann–Whitney *t* test was used to evaluate the difference between the groups. The Kurskal–Wallis test was performed to distinguish more than two groups. For sensitivity and specificity analysis and for comparison of assays, receiver operating characteristic (ROC) curves were constructed. Statistical analyses were performed using the Graph Pad Prism 5.0 (Graph Pad Software, La Jolla, CA, USA). The level of significance was set at *P* < 0.05.

## Results

**Fig. 1:**
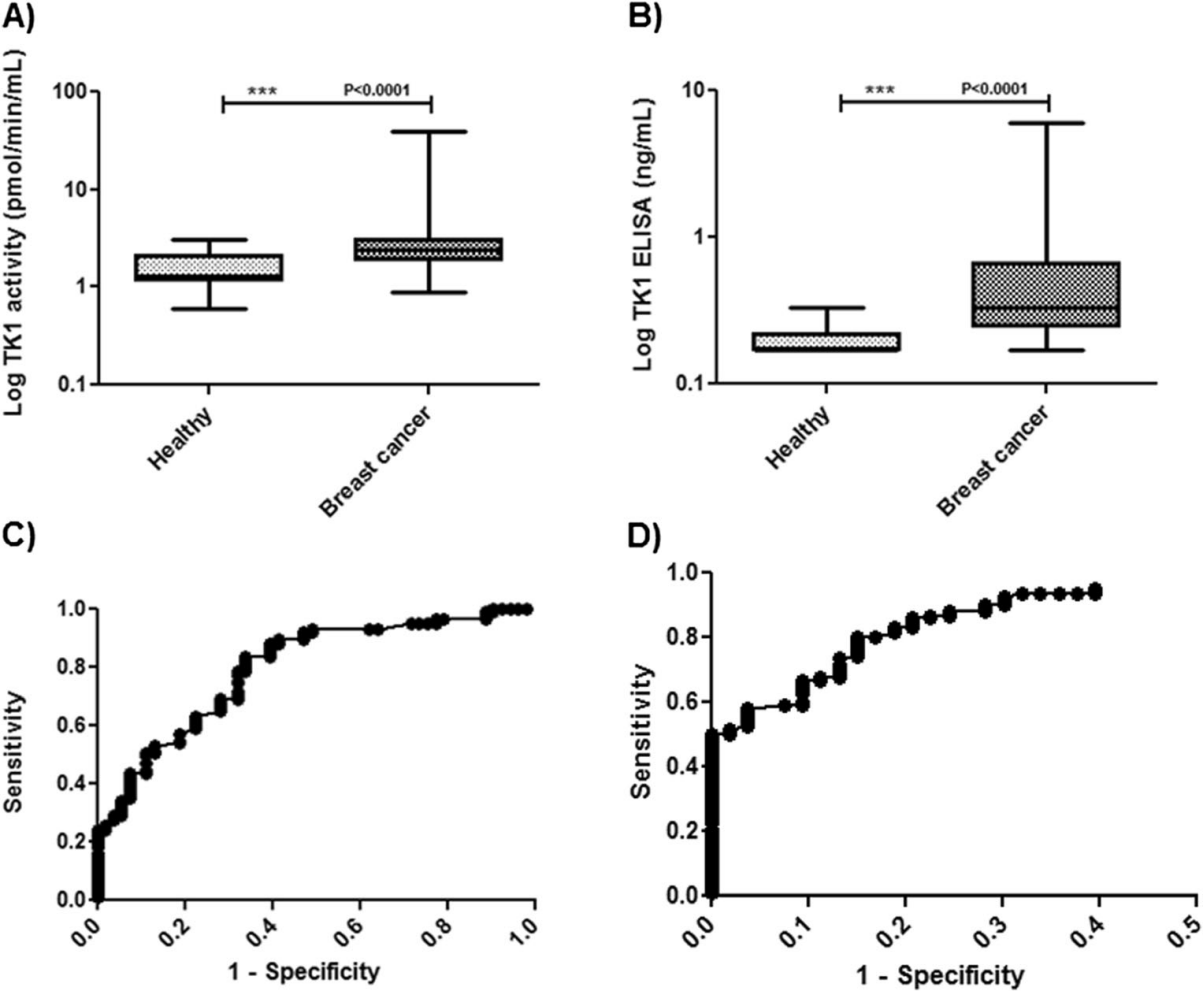
TK1 activity and protein levels in sera from healthy and from the entire breast cancer groups. **a** Log TK1 activity levels in sera from breast patients and healthy individuals. **b** Log TK1 protein levels in sera from healthy and breast cancer patients. *Error bars* denote maximum and minimum values. The receiver operating characteristic (*ROC*) curves **c** TK1 activity assay. **d** TK 210 ELISA for breast cancer patients in comparison with healthy individuals

Serum samples from 53 healthy individuals and 124 breast cancer patients were analyzed for serum TK1 activity and TK1 protein levels, as described in the “Materials and methods” section. The LOD was 0.17 ng/ml, and within-run CVs were less than <10 % for the TK 210 ELISA. CV between the runs was <20 %, and in studies of recovery and linearity on dilution-measured values ranged from 91 to 105 % of expected at concentrations of 0.22–22 ng/ml. In healthy, TK1 activity values ranged from 0.6 to 3.1 pmol/min/ml (mean ± SD = 1.6 ± 0.6) and in breast cancer patients, the STK1 activity levels were significantly higher compared to the healthy group (Fig. 1a), in the range of 0.9 to 48 pmol/min/ml (mean ± SD = 4.5 ± 8.3) with a median value of 2.5 pmol/min/ml. Many of the sera from healthy persons had TK1 protein levels at or below the detection limit (0.17 ng/ml) and 40 % of them had detectable TK1 protein levels in the range of 0.17 to 0.33 ng/ml (the estimated mean ± SD = 0.2 ± 0.05 using the LOD value for those below this value). Significantly higher serum TK1 protein levels were found in sera from breast cancer patients compared to healthy, and the protein concentration ranged from 0.17 to 9.9 ng/ml (mean ± SD = 1.0 ± 1.9) (Fig. 1b). TK1 activity and TK1 protein levels in healthy as well as breast cancer patients did not show any significant correlation with the age of the individuals (*P* = 0.51, *P* = 0.41, *P* = 0.56, *P* = 0.44). However, significant correlations were found between the serum TK1 activity and TK1 protein levels in healthy (*rs* = 0.72, *P* < 0.0001) and breast cancer patients (*rs* = 0.60, *P* < 0.0001). Overall, TK-Liaison assay had significant correlation with the ^3^[H]-dThd phosphorylation assay (*rs* = 0.75, *P* < 0.0001) as well as with TK 210 ELISA (*rs* = 0.58, *P* < 0.0001).

### TK1 activity, TK1 protein, and CA 15-3 levels in sera from patients with breast cancer

The performance of the TK1 activity assay, TK 210 ELISA, and CA 15-3 with sera from patients with all stages of breast cancer were evaluated by constructing ROC curves. The results of these analyses are shown in Table 1 and Fig. 1. The TK1 activity assay showed an area under the curve (AUC) of 0.79 using a cut-off value of 3.0 pmol/min/ml, the sensitivity was 0.26, and the specificity of 0.96 (Fig. 1c). The TK 210 ELISA, however, had an AUC of 0.90, *P* < 0.0001, and at a cut-off value of 0.32 ng/ml, the sensitivity obtained was 0.50 and the specificity 0.98 (Table 1) (Fig. 1d). The CA 15-3 values in the breast cancer sera showed a broad range from 6 to 716 U/ml with a median of 24 U/ml (mean ± SD = 67 ± 115). Further analysis compared the CA 15-3 and serum TK1 activity and protein values; a significant correlation was found between CA 15-3 and TK1 protein levels (*rs* = 0.33, *P* = 0.0022) for the entire breast cancer group but this was not observed with TK1 activity levels (*P* = 0.14).

**Table 1:** Results from the ROC curve analysis with the different assays

Group	AUC	Sensitivity [95 % CI]	Specificity [95 % CI]
Breast cancer vs healthy			
TK1 activity assay	0.79	0.26 [0.19–0.35]	0.96 [0.89–0.97]
TK 210 ELISA	0.90	0.50 [0.42–0.59]	0.98 [0.89–0.99]
T1 vs healthy			
TK1 activity assay	0.76	0.14 [0.05–0.30]	0.96 [0.86–0.97]
TK 210 ELISA	0.88	0.34 [0.19–0.52]	0.98 [0.89–0.99]
CA 15-3	0.65	0.20 [0.08–0.36]	0.98 [0.89–0.99]
TK 210 ELISA + CA15-3	0.76	0.40 [0.32–0.56]	0.98 [0.89–0.99]
T2 vs healthy			
TK1 activity assay	0.86	0.39 [0.26–0.53]	0.96 [0.86–0.97]
TK 210 ELISA	0.92	0.61 [0.49–0.71]	0.98 [0.88–0.99]
CA 15-3	0.82	0.63 [0.48–0.75]	0.98 [0.89–0.99]
TK 210 ELISA + CA15-3	0.94	0.76 [0.64–0.84]	0.98 [0.89–0.99]

Cut-off values for TK1 activity assay is >3.0 pmol/min/ml, for TK 210 ELISA is >0.32 ng/ml, and for CA 15-3 >29 U/ml to determine the sensitivity and specificity

*AUC* area under curve, *CI* confidence interval

### TK1 activity, protein, and CA 15-3 levels in relation to T1 to T4 stage

**Fig. 2:**
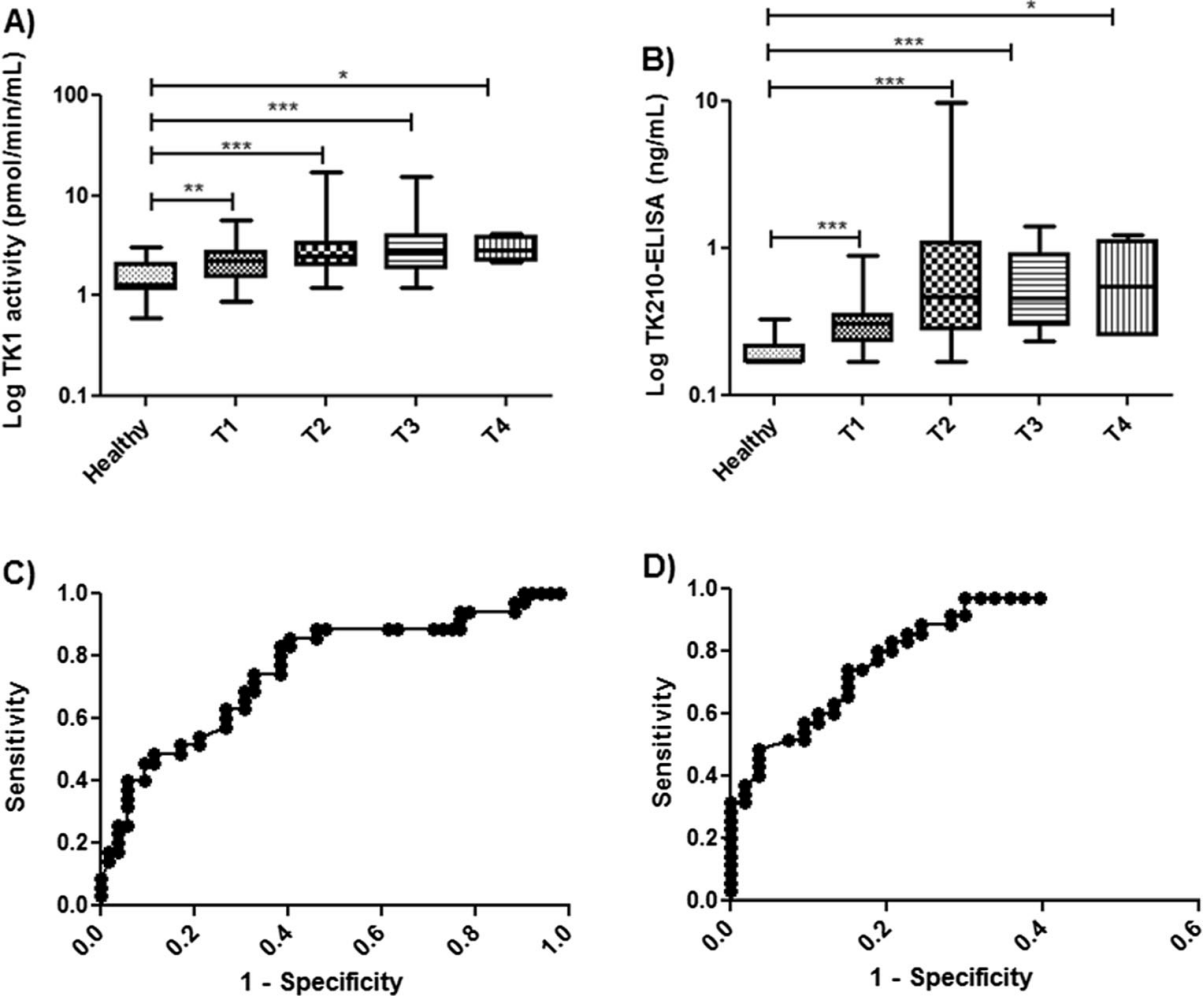
TK1 activity and protein levels in breast cancer sera based on stage (T1–T4). **a** Log TK1 activity levels in sera from breast cancer patients based on T stage (T1–T4) and healthy individuals. **b** Log TK 210 ELISA in sera from healthy and breast cancer patients (T1–T4). *Error bars* denote maximum and minimum values. The ROC curveanalysis for **c** TK1 activity assay. **d** TK 210 ELISA for T1 breast cancer patients in comparison with healthy individuals

The median TK1 activity values were somewhat higher in T1 (2.2 pmol/min/ml) compared to T4 stage disease (2.8 pmol/min/ml), however, the TK1 activity levels in sera from patients at all these stages significantly differ from those in healthy individuals (Fig. 2a). The TK1 protein levels increased more clearly but overall followed a similar pattern with regard to tumor stage. The median values were 0.33 and 0.55 ng/ml in T1 and T4 patients, respectively (Fig. 2b). Furthermore, no correlations were found between TK1 activity, TK1 protein, and CA 15-3 values in T1 patients. The ROC curves for TK1 activity in T1 patients showed an AUC of 0.76 (Fig. 2c), the sensitivity was 0.14 with a specificity of 0.96 (Table 1), while the TK 210 ELISA showed a significantly higher AUC (0.88) as well as a sensitivity of 0.34 and a specificity of 0.98 (Fig. 2d). CA 15-3 demonstrated slightly higher sensitivity compared to the TK1 activity assay, but it was less than TK1 ELISA (Table 1). These results indicate that TK1 ELISA can detect early stage breast cancer better than the TK1 activity and CA 15-3 assays.

**Fig. 3:**
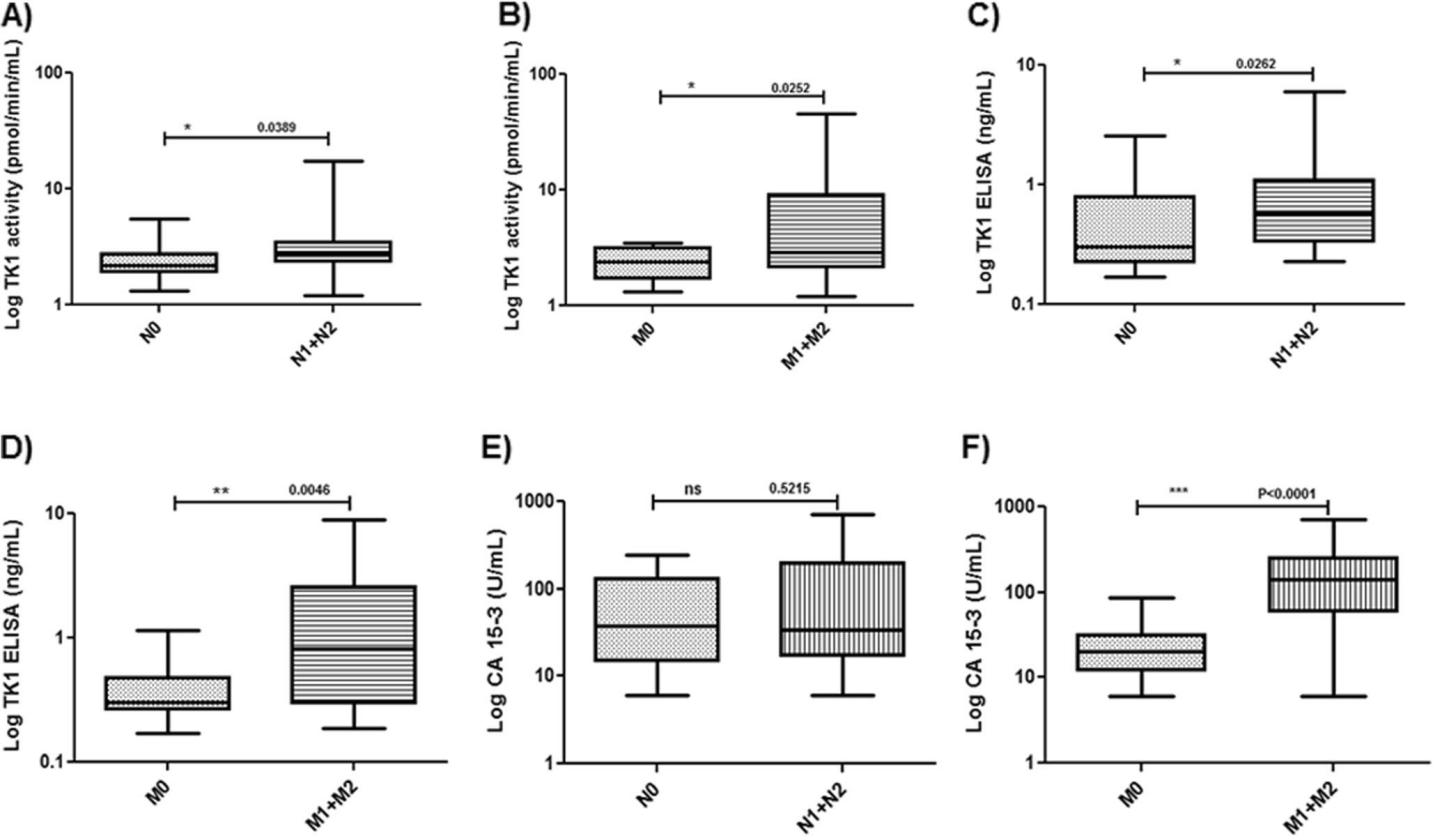
TK1 activity, TK 210 ELISA, and CA 15-3 values in T2 patients. **a** Log TK1 activity levels in T2 patients with lymph node involvement (N1 + N2) without nodal involvement (N0). **b** Log TK1 activity levels in T2 patients with metastasis (M1 + M2) without metastasis (M0). **c** Log TK 210 ELISA values in T2 patients with lymph node involvement (N1 + N2) without nodal involvement (N0). **d** Log TK 210 ELISA values in T2 patients with metastasis (M1 + M2) without metastasis (M0). **e** Log CA 15-3 levels in T2 patients with lymph node involvement (N1 + N2) without nodal involvement (N0). **f** Log CA 15-3 levels in T2 patients with metastasis (M1 + M2) without metastasis (M0)

In T2 patients, TK1 activity, TK 210 ELISA, and CA 15-3 levels were significantly higher compared to those in T1 patients (Table 2) (Fig. 3). Furthermore, TK1 activity and TK protein levels were increased in patients with lymph node involvement and metastatic disease compared to those without such complications (Fig. 3a–d). In the case of CA 15-3, only metastatic patients had significantly higher CA 15-3 levels (Fig. 3e, f, Table 2). We also found significant correlations between the three assays, i.e., TK1 activity and TK 210 ELISA (*r* = 0.65, *P* < 0.0001), TK1 activity and CA 15-3 (*r* = 0.41, *P* = 0.0028), and TK 210 ELISA and CA 15-3 (*r* = 0.46, *P* = 0.0005).

**Table 2:** Fraction of breast cancer patients with positive TK1 activity, TK 210 ELISA, and CA 15-3 values in relation to clinical and pathologic parameters

Parameter	No. of patients	TK1 activity (pmol/min/ml)	TK1 ELISA (ng/ml)	CA 15-3 (U/ml)
		Patient no. (% >3.0)	Patient no. (% >0.32)	Patient no. (% >29)
Stage				
T1	35	5 (14)	12 (34)	7 (20)
T2	54	22 (40)	33 (61)	34 (63)
T3	12	5 (38)	8 (67)	7 (58)
T4	4	2 (50)	2 (50)	3 (75)
Subsets in T1				
LN−	23	5 (21.7)	8 (34.7)	3 (13)
LN+	12	1 (8.25)	4 (33.3)	4 (33)
M−	32	5 (15.6)	10 (31.2)	6 (19)
M+	3	1 (33.3)	2 (66.7)	2 (66.7)
Subsets in T2				
LN−	24	8 (30)	14 (58.3)	15 (62.5)
LN+	30	14 (46.6)	21 (70)	18 (60)
M−	23	7 (24.1)	13 (44.8)	8 (34.7)
M+	27	13 (48.1)	20 (74)	24 (88)
Histological grade				
DCIS	6	1 (16.6)	5 (83.3)	5 (83.3)
ID/L	9	6 (66.6)	7 (77.7)	8 (88.8)
IDC	26	11 (38.4)	18 (69.2)	20 (74)

*T* tumor size, *LN* lymph node involvement, *M* systemic metastasis, *DCIS* ductal in situ carcinoma, *ID*/*L* invasive ductal/ lobular carcinoma, *IDC* invasive ductal carcinoma

**Fig. 4:**
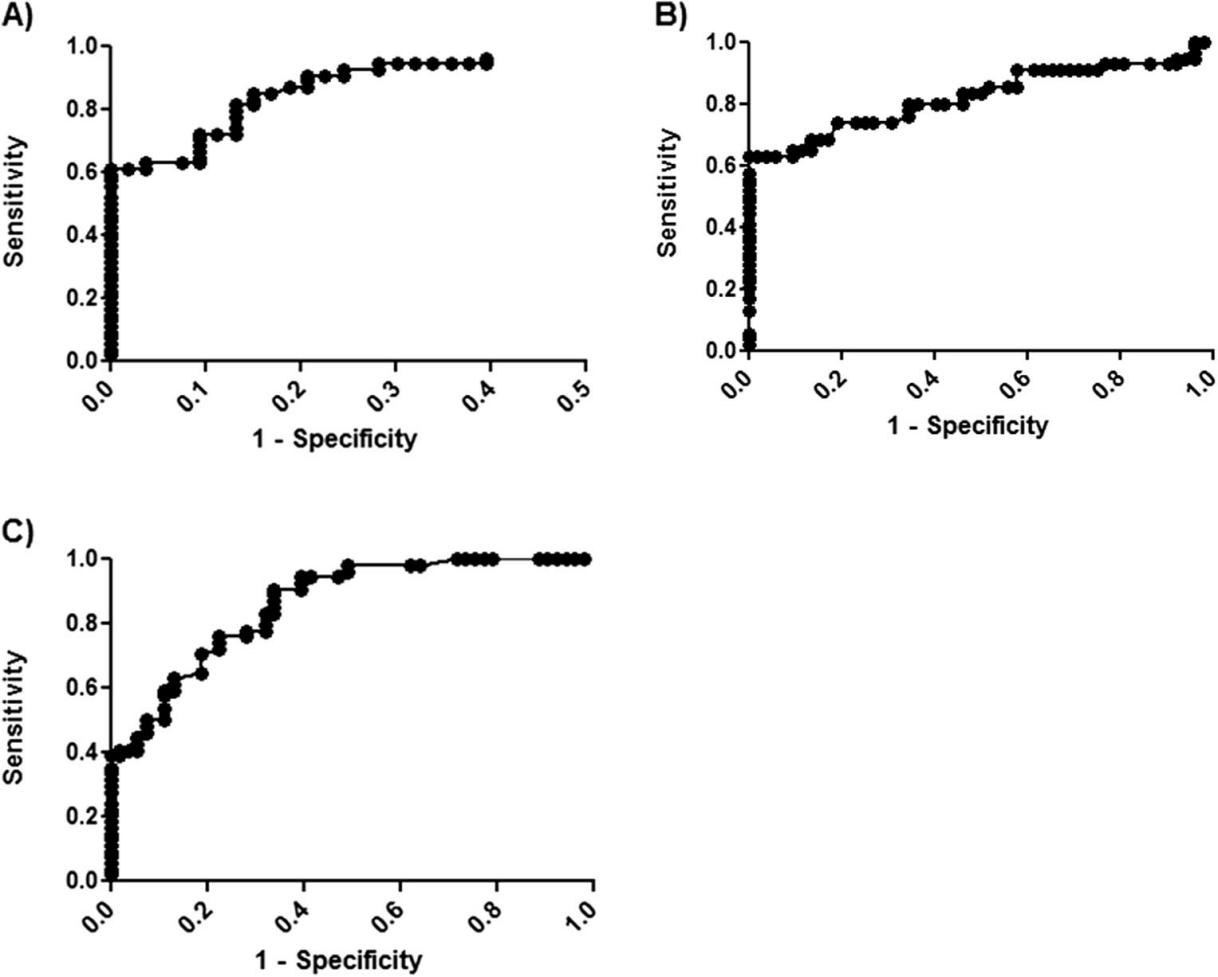
ROC curve analysis for **a** STK 210 ELISA levels, **b** CA 15-3 levels, and **c** STK1 assays with T2 breast cancer patients and healthy individuals

In ROC curve analysis, the TK 210 ELISA as well as CA 15-3 (Fig. 4a, b) showed superior sensitivity compared to TK1 activity assay (Fig. 4c) in patients with T2 stage breast cancer. However, the combination of TK 210 ELISA and CA 15-3 increased the sensitivity significantly from 0.6 to 0.76 and the AUC from 0.92 to 0.94 (Table 1, supplement Fig 1). These results strongly indicate that the two assays can complement each other, thereby increasing the clinical usefulness of these assays. In 41 out of 124 patients with known histological grading (DCIS, ID/L, and IDC), the DCIS and ID/L patients apparently show higher serum TK1 protein as well as CA 15-3 levels compared to the other types (Table 2). Taken together, these results indicate that TK 210 ELISA should be a valuable complement to the in vitro diagnostic procedures applied for breast cancer patients.

## Discussion

Proliferation in tumor cells correlates with high levels of TK1 since this enzyme plays an important role in pyrimidine deoxynucleotide synthesis. Because of this characteristic, TK1 has been used as a proliferation marker in lymphomas and leukemias in human medicine for many years [10–12]. Several commercial assays are available to measure the TK1 activity levels in different malignancies, such as e.g., TK-REA, TK-Liaison, and Divitum [10, 29, 30]. Even though TK1 activity levels show a correlation with the stage of breast cancer disease, the overall sensitivity of the assays is rather low i.e., less than 35 % [30–32]. This limits the clinical use of TK1 activity assays in breast cancer patients.

Development of antibodies against the C-terminal region of TK1 provides an alternative way to determine TK1, and in this case, it is the TK1 protein levels that are measured [18]. Previous studies with a dot blot assay showed that when the TK1 protein levels were determined they were significantly higher in sera from patients with many different types of solid tumors [20, 21]. A recent study with an immunoaffinity assay demonstrated that TK1 protein levels are significantly increased in sera from patients with early stage breast and prostate tumor disease, while the TK1 activity was not. A reason for this could be that there was a larger proportion of inactive TK1 in serum from patients with solid tumors compared to those seen in sera from leukemia patients [24]. Therefore the development of an ELISA based assay, which could determine both the active and inactive TK1 protein in sera from cancer patients should be a great advantage in in-vitro diagnostics.

The first clinical study with commercial TK1 (AroCell AB, Uppsala, Sweden) sandwich ELISA was published in 2009 with a combination of monoclonal and polyclonal antibody made against peptides from the C-terminal region of TK1 [33]. However, this ELISA had relatively low sensitivity and specificity and relied on polyclonal antibodies, making robust production of the test complicated. The new version of TK 210 ELISA from AroCell AB has been developed based on two monoclonal antibodies. It can overcome the problems with polyclonal antibodies, but it also increases the sensitivity of the assay as demonstrated in this study. Some earlier studies with other TK1 ELISA assays have been published [25, 26] but the details about the antibodies are not reported. An attempt to develop a direct ELISA based on one monoclonal antibody for detection of early stage lung cancer has been published [27]. However, the levels of serum TK1 reported are several orders higher than those described here and the commercial availability of this assay is unknown [27].

To the best of our knowledge, this study is the first to demonstrate the performance of a fully developed TK 210 ELISA, which is commercially available. The results reported here strongly suggest that the TK 210 ELISA is able to detect a significant differences in the levels of the TK1 protein in sera from breast cancer patients in the T1, T2, and T3 stages compared to healthy. The results with the TK activity assay gave over all similar results, and there was a correlation between the two assays, but the sensitivity of the activity assay was approximately half of the TK 210 ELISA. Significant differences were observed between the serum TK1 levels in the group of patients with lymph node involvement and metastatic disease compared to those without, suggesting that the TK 210 ELISA can be used for prognosis as well as monitoring of breast cancer both in early and later stages of the disease.

Several studies have been done to determine the clinical value of CA 15-3 as a biomarker in breast cancer. CA 15-3 has low sensitivity (10 %) for detection of early stage tumors [34], but is widely used for monitoring therapy of patients with advanced stages of breast cancer [35, 36]. The overall sensitivity of CA 15-3 assay for breast cancer is in the range from 20 to 50 % [34, 37, 38]. In metastatic patients, a combination of CA 15-3 with CEA and HER 2 increased the sensitivity to 64 % [39], and a recent study claimed that the combination of CEA, CA 15-3, and TK1 protein increased the sensitivity to 90 % [25]. In the present study, we found positive correlations between TK1 activity and TK1 protein values with the CA 15-3 values, especially in the case of T2 patients. The combination of TK 210 ELISA and CA 15-3 biomarkers increased the sensitivity from 0.61 and 0.63 respectively, of each alone to 0.76 when the two marker were combined. Thus, the results from this study strongly suggest that a combination of TK 210 ELISA with CA 15-3 will be highly informative as they apparently provide complementary information. Further clinical evaluation of TK 210 ELISA in breast cancer management, in larger case-control clinical studies seems therefore highly motivated.

## Electronic supplementary material

Below is the link to the electronic supplementary material.

Supplement Fig 1 ROC curve analysis of TK 210 ELISA levels (FX) and CA 15-3 levels (FX) and the combination of TK 210 ELISA and CA 15-3 (FX) with T2 breast cancer patient and healthy individuals (GIF 272 kb)

High resolution image (TIFF 212 kb)
