# Supplementary figures and images for: A clinical evaluation of the TK 210 ELISA in sera from breast cancer patients demonstrates high sensitivity and specificity in all stages of disease
Source: Tumour Biol. 2016 Apr 14;37(9):11937–45. doi: 10.1007/s13277-016-5024-z (PMC5080325; doi:10.1007/s13277-016-5024-z)

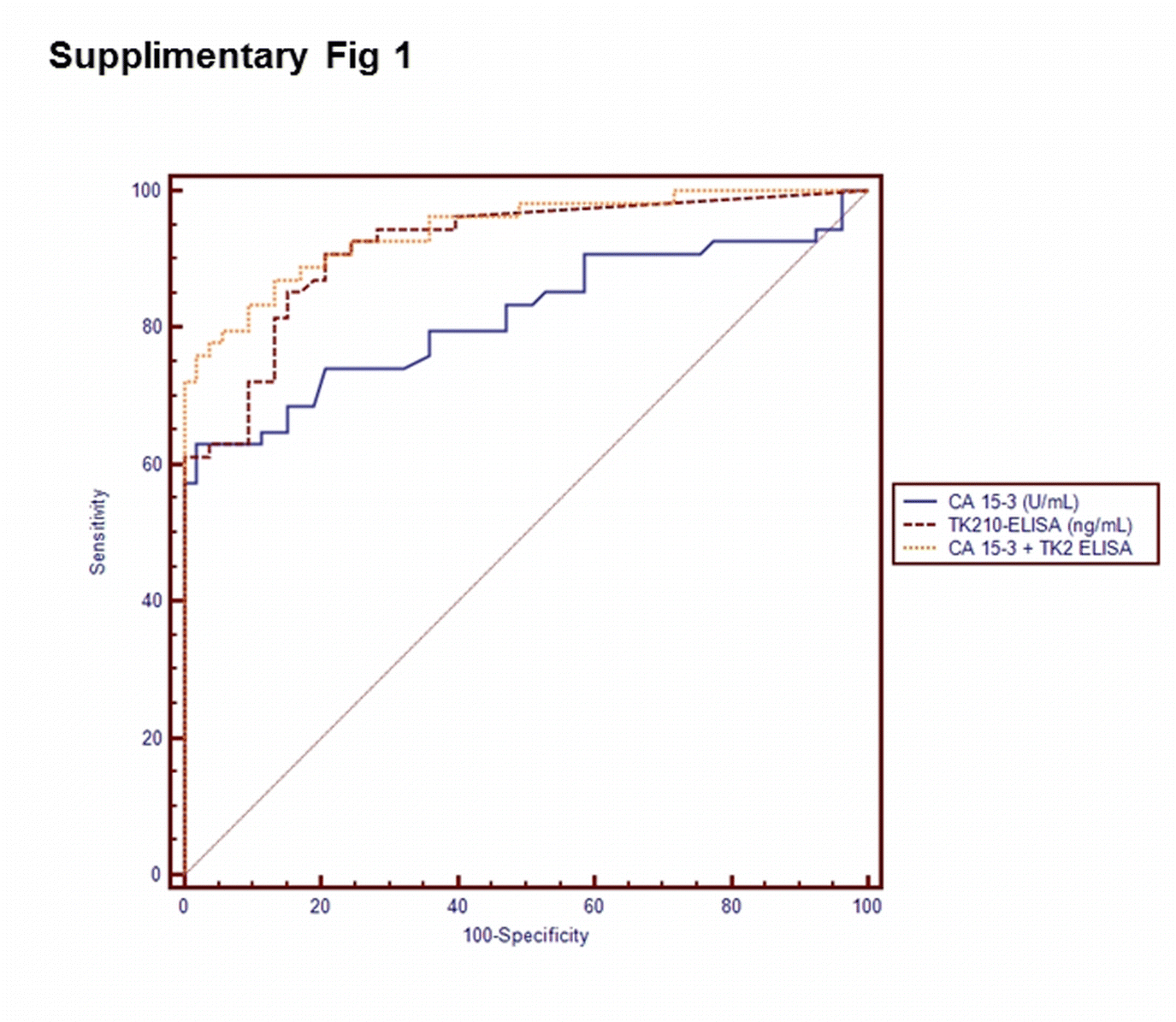

Supplement: Supplementary file 1 — ROC curve analysis of TK 210 ELISA levels (FX) and CA 15-3 levels (FX) and the combination of TK 210 ELISA and CA 15-3 (FX) with T2 breast cancer patient and healthy individuals (GIF 272 kb) [file 13277_2016_5024_Fig5_ESM.gif]

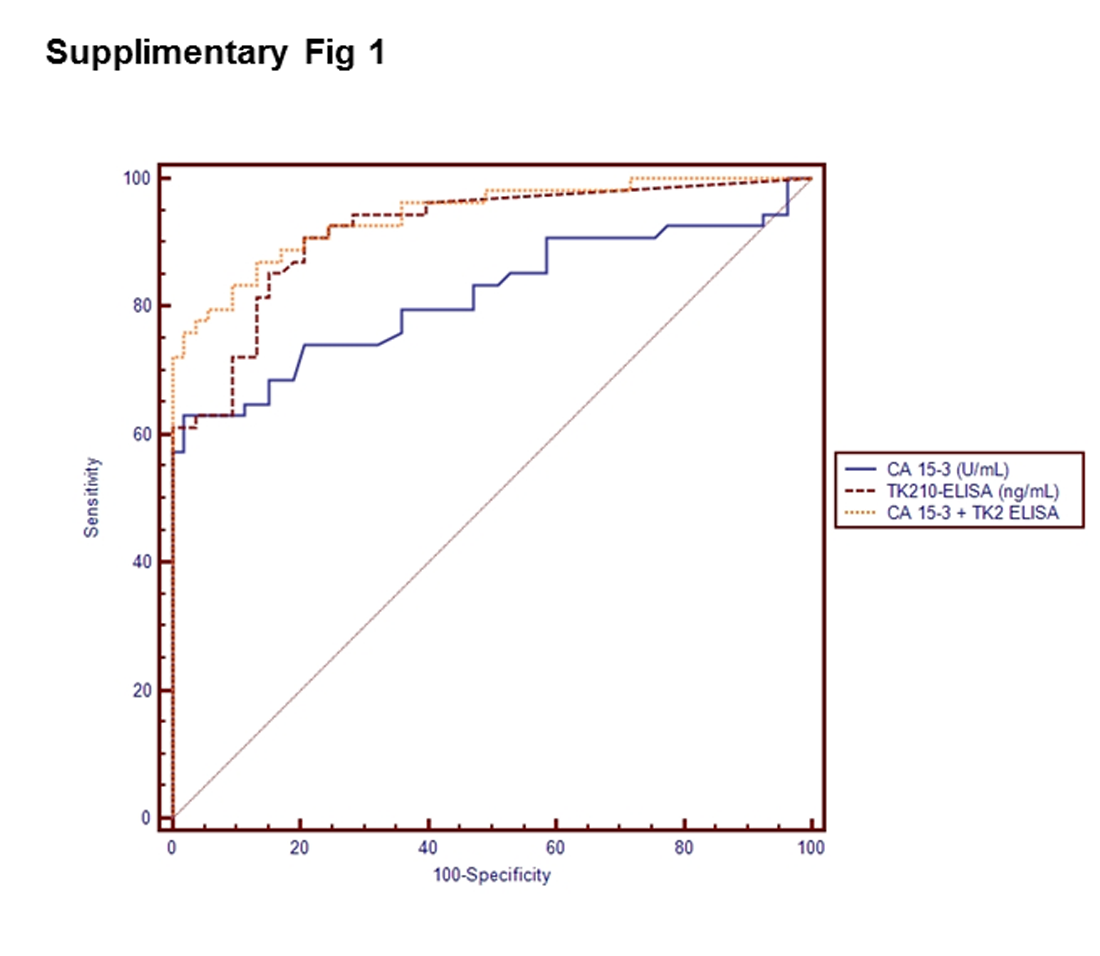

Supplement: Supplementary file 2 — High resolution image (TIFF 212 kb) [file 13277_2016_5024_MOESM1_ESM.tiff]
